# Revealing the *Salmo salar* NLRP3 Inflammasome: Insights from Structural Modeling and Transcriptome Analysis

**DOI:** 10.3390/ijms241914556

**Published:** 2023-09-26

**Authors:** Waldo Acevedo, Rodrigo Morán-Figueroa, Luis Vargas-Chacoff, Francisco J. Morera, Juan Pablo Pontigo

**Affiliations:** 1 Biological Chemistry Laboratory, Institute of Chemistry, Faculty of Science, Pontificia Universidad Católica de Valparaíso, Valparaiso 2373223, Chile; waldo.acevedo@pucv.cl; 2 Escuela de Medicina Veterinaria, Facultad de Agronomía y Sistemas Naturales, Pontificia Universidad Católica de Chile, Santiago 7810000, Chile; rodrigo.moran@ug.uchile.cl; 3 Escuela de Medicina Veterinaria, Facultad de Ciencias Biológicas, Pontificia Universidad Católica de Chile, Santiago 7810000, Chile; 4 Escuela de Medicina Veterinaria, Facultad de Medicina, Pontificia Universidad Católica de Chile, Santiago 7810000, Chile; 5 Institute of Marine Sciences and Limnology, Faculty of Sciences, Universidad Austral de Chile, Valdivia 5110566, Chile; luis.vargas@uach.cl; 6 IDEAL Research Center for Dynamics of High Latitude Marine Ecosystems, Universidad Austral de Chile, Valdivia 5110566, Chile; 7 Millennium Institute Biodiversity of Antarctic and Subantarctic Ecosystems, BASE, University Austral of Chile, Valdivia 5090000, Chile; 8 Integrative Biology Group, Valdivia 5110566, Chile; 9 Laboratorio Institucional de Investigación, Facultad Ciencias de la Naturaleza, Medicina Veterinaria, Universidad San Sebastián, Lago Panguipulli 1390, Puerto Montt 5090000, Chile

**Keywords:** NLRP3, inflammasome, innate immune response, *Atlantic salmon*, molecular docking, transcriptomics

## Abstract

The NLRP3, one of the most heavily studied inflammasome-related proteins in mammals, remains inadequately characterized in *Atlantic salmon* (*Salmo salar*), despite the significant commercial importance of this salmonid. The NLRP3 inflammasome is composed of the NLRP3 protein, which is associated with procaspase-1 via an adapter molecule known as ASC. This work aims to characterize the *Salmo salar* NLRP3 inflammasome through in silico structural modeling, functional transcript expression determination in the SHK-1 cell line in vitro, and a transcriptome analysis on *Atlantic salmon*. The molecular docking results suggested a similar arrangement of the ternary complex between NLRP3, ASC, and caspase-1 in both the *Atlantic salmon* and the mammalian NLRP3 inflammasomes. Moreover, the expression results confirmed the functionality of the SsNLRP3 inflammasome in the SHK-1 cells, as evidenced by the lipopolysaccharide-induced increase in the transcription of genes involved in inflammasome activation, including ASC and NLRP3. Additionally, the transcriptome results revealed that most of the inflammasome-related genes, including ASC, NLRP3, and caspase-1, were down-regulated in the *Atlantic salmon* following its adaptation to seawater (also known as parr–smolt transformation). This is correlated with a temporary detrimental effected on the immune system. Collectively, these findings offer novel insights into the evolutionarily conserved role of NLRP3.

## 1. Introduction

Numerous immune-system-related genes across various teleost fish species have been notated in recent years. Although they are similar to those found in mammals, their biological functions are still undetermined [1]. Mammals and fish diverged from an ocean-dwelling common ancestor 450 million years ago. Surprisingly, there is still little background regarding how different the immune-system-signaling pathways are between them [2]. In general, when comparing the immune systems of fish with those of mammals, fish innate immunity seems to be highly evolved, with more significant potential and functionality, while the adaptive immunity of fish appears to be less sophisticated than that of mammals [3,4]. Innate immunity is the teleost fish’s first line of defense [4,5]. Pattern-recognition receptors (PRRs) play a vital role in the initiation of this innate immunity [6,7] because these receptors are activated in response to harmful stimuli, such as invading pathogens, dead cells, and environmental irritants [8]. These PRRs are intracellular multiprotein complexes that specialize in the recognition of pathogen-associated molecular patterns (PAMP) and damage-associated molecular patterns (DAMP) [9,10,11]. These recognition mechanisms trigger an inflammatory response to defend against microbial infections and repair damaged tissues by activating inflammasomes [6]. Inflammasomes are multimolecular complexes that transduce signals detected by specific cytosolic proteins in the NLRP family and produce, as a response, the activation of caspase-1 or other caspases with inflammatory functions [6,12,13,14,15].

In teleosts, inflammasomes are divided into four different subfamilies, according to the structural organizations of their domains [16]: (i) the NOD-like receptor (NLR) subfamily with an acidic domain (NLR-A), which is similar to the mammalian nucleotide-binding oligomerization domains (NODs); (ii) the NLR subfamily, with a BIR domain (NLR-B), which bears a resemblance to the mammalian NACHT-, LRR-, and PYD-domain-containing proteins (NALPs); (iii) the NLR subfamily, with a CARD domain (NLR-C), a subfamily unique to bony fish; and (iv) the NLR subfamily, with a pyrin domain (NLRP), which has been widely described in mammals, but so far has only been identified in the zebrafish and the Japanese flounder [17]. Recent studies have characterized some NLRP3 inflammasome components, such as apoptosis-associated speck-like protein containing CARD (ASC), caspase-1, interleukin (IL)-1b, and NLRP3 in the Japanese flounder (*Paralichthys olivaceus)*, the green-spotted puffer (*Tetraodon nigroviridis)*, and the zebrafish (*Danio rerio*), [18,19,20,21].

The NLRP3 is one of the most studied inflammasome-related proteins in mammals [22], but its characterization in the *Atlantic salmon* remains incomplete. The NLRP3 inflammasomes are characterized by containing NLRP3 protein associated with caspase-1 through the adapter known as ASC [23]. Structurally, the NLRP3 protein has three domains: (i) a central NACHT domain coupled with a C-terminal leucine-rich repeat domain (LRR domain); (ii) a NACHT domain exhibiting ATPase activity; and (iii) an N-terminal pyrin domain (PYD) [24].

On the other hand, caspase-1 contains an N-terminal recruiter domain (CARD), a central large catalytic domain (p20), and a C-terminal small catalytic subunit domain (p10). Caspase-1 can associate with ASC protein through CARD-CARD2 binding, whereas NLRP3 interacts with ASC through homotypic PYD–PYD binding [25,26,27]. the recruitment of ASC allows caspase-1 to self-cleave and, thus, release p20–p10 subunits [28]. This heterotetramer subsequently cleaves pro-IL-1β/pro-IL-18 cytokines [29,30].

The ASC plays a critical role in caspase-1 activation through two mechanisms: (i) by serving as a molecular linker between PYD- and CARD-containing signaling molecules within the inflammasome (cytosolic PRR multiprotein complex); or (ii) through the oligomerization of ASC monomers in a cytosolic assembly, named pyroptosome [15,31]. This complex leads to a cell-death mechanism mediated by caspase-1 activation, through which macrophages die rapidly after being infected with intracellular pathogens, facilitating the elimination of the pathogens [32].

This work aims to characterize the *Salmo salar* NLRP3 (SsNLRP3) inflammasome through in silico structural modeling, in vitro functional assays, and transcriptomic analysis. To this end, we performed a comparative analysis of the NLRP3 sequences in the *Atlantic salmon* and in humans through sequence alignments. Subsequently, we built a homology model of three *Atlantic salmon*-inflammasome components—NLRP3, ASC, and procaspase-1—and predicted their interactions.

Subsequently, we performed a transcript-expression analysis using qPCR in the SHK-1 cell line stimulated with LPS at different times to evaluate the changes in the expression levels of inflammasome-related genes. Finally, we analyzed expression changes in the inflammasome-pathway-related genes in *Atlantic salmon* head kidneys using transcriptomic data from the parr and smolt stages of the freshwater phase in the *Atlantic salmon* life cycle. Despite numerous studies on mammals, the characterization of NLRP3 in lower vertebrates, especially teleosts, remains largely incomplete. Our study is the first to reveal previously uncharacterized components of NLRP3 in *Atlantic salmon*, providing novel insights into the evolutionary role of NLRP3. This lays the groundwork for future studies on its role during bacterial infection and the overall innate immune response of fish.

## 2. Results

### 2.1. Phylogenetic Analysis

**Figure 1:**
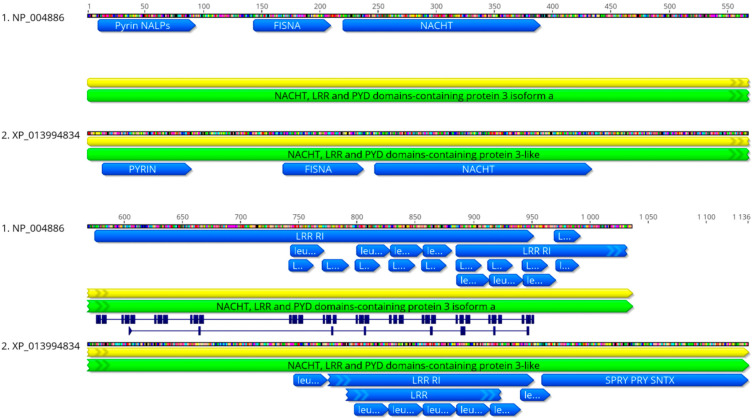
Comparison of functional domains of *Atlantic salmon* NLRP3 and human NLRP3. Pyrin, FISNA, and NACHT domains and leucine repeats are shown in blue in *Atlantic salmon* (XP_013994834) and humans (NP_004886).

The phylogenetic analysis of the amino acid sequence for the SsNLRP3 from *Atlantic salmon* with different species was carried out using the Bayesian method. The SsNLRP3 sequence of *Atlantic salmon* is related to the taxonomic type of bony fish together with the sequences of *Oncorhynchus mykiss*, *Takifugu rubripes,* and *Danio rerio*, a little more distantly from *Perca flavecens* and *Larimichthys crocea,* and not very distant from mammals such as *Homo sapiens, Rattus norvergicus,* and *Macaca mulatta* (Figure S2). The domains in the *Atlantic salmon* NLRP3 (XP_013994834) were shown to have fewer leucine repeats and an extra Spry Pry sntx domain that does not have human NLRP3 (NP_004886), presenting in common the PYRIN, FISNA, and NATCH domains (Figure 1).

### 2.2. Homology Modeling of SsNLRP3, SsASC, and SsCaspase-1

**Figure 2:**
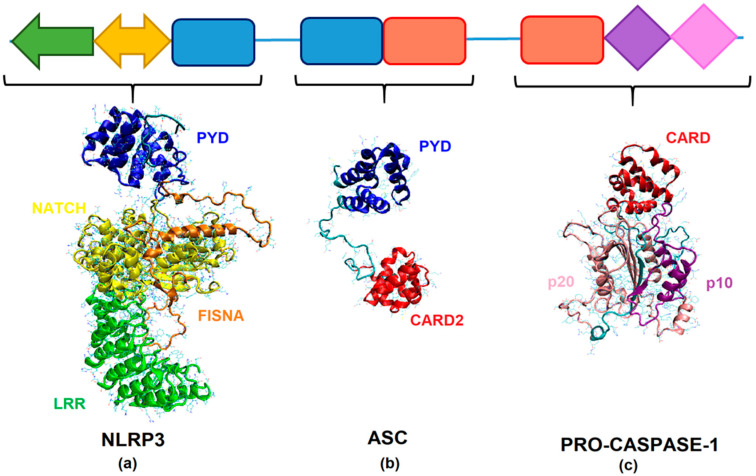
Three-dimensional structure of *Atlantic salmon* (**a**) NLRP3, (**b**) ASC, and (**c**) Caspase-1 at atomistic level of detail. The protein was rendered in the form of new cartoons. Domains are colored as follows: PYD—blue, FISNA—orange, NATCH—yellow, LRR—green, CARD and CARD2—red, p20—pink and p30—purple.

The templates for the homology modeling of the SsNLRP3, SsASC, and SsCaspase-1 were selected based on both the sequence identity and the e-value after using BLAST. Chain A of the human NLRP3 (PDB entry 6NPY) and human ASC (PDB entry 2KN6) showed sequence identities of 27.43% and 32.17% and e-values of 1 × 10^−70^ and 4 × 10^−13^ against the SsNLRP3 and SsASC, respectively. Chain A of the human caspase-1 CARD (PDB entry 5FNA) and the human inhibitor of interleukin-1 beta generation (PDB entry 1DGN) showed sequence identities of 44.30% and 44.32% and e-values of 4 × 10^−14^ and 9 × 10^−18^ against caspase-1 CARD2, respectively, whereas chain A of the human procaspase-1 zymogen domain (PDB entry 3E4C) showed a sequence identity of 48.86% and an e-value of 1 × 10^−75^ against the p20 and p10 subunits of ssCaspase-1. Figure 2a–c illustrate the three-dimensional structure of SsNLRP3, SsASC, and SsCaspase-1.

The Ramachandran plots (Supplementary Figure S3) showed that 82.8% of the residues are in favored regions, 16.2% in allowed regions, and 1.0% in outlier regions for SsNLRP3; 92.9% of the residues are in favored regions, 6.0% in allowed regions, and 1.1% in outlier regions for SsASC; and 86.0% of the residues are in favored regions, 13.7% in allowed regions, and 0.3% in outlier regions for SsCaspase-1. The PROCHECK analysis of the SsNLRP3, SsASC, and SsCaspase-1 models yielded overall average G-factors of −0.21, −0.05, and −0.28, respectively. The Q-MEAN values were −5.05, −3.79, and −3.34 for the SsNLRP3, SsASC, and SsCaspase-1, respectively, suggesting that the resulting models were sufficiently close to a set of experimental protein structures from the PDB database.

### 2.3. In Silico Analysis of the Interaction between NLRP3, ASC, and Caspase-1

**Figure 3:**
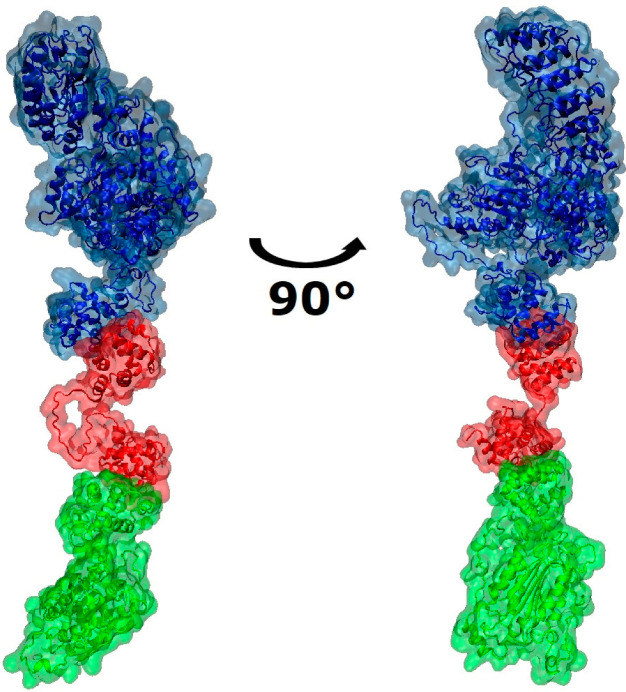
Visualization of binding interface between the components of *Atlantic salmon* inflammasome. Components are colored as follows: ssNLRP3—blue, ssASC—red and ssCaspase-1—green.

Figure 3 depicts the potential binding sites and poses of the *Atlantic salmon*’s inflammasome components, which is the NLRP3 pyrin domain’s binding to the ASC pyrin domain and the ASC CARD domain’s binding to the caspase-1 CARD2 domain. In addition, both hydrogen bonding and hydrophobic contacts (Table 1, Supplementary Figures S4 and S5) govern the interactions of binding-interface amino acids between inflammasome components.

### 2.4. Lipopolysaccharide (LPS) Modulates the Transcription of Genes Involved in the Activation of the Inflammasome

**Figure 4:**
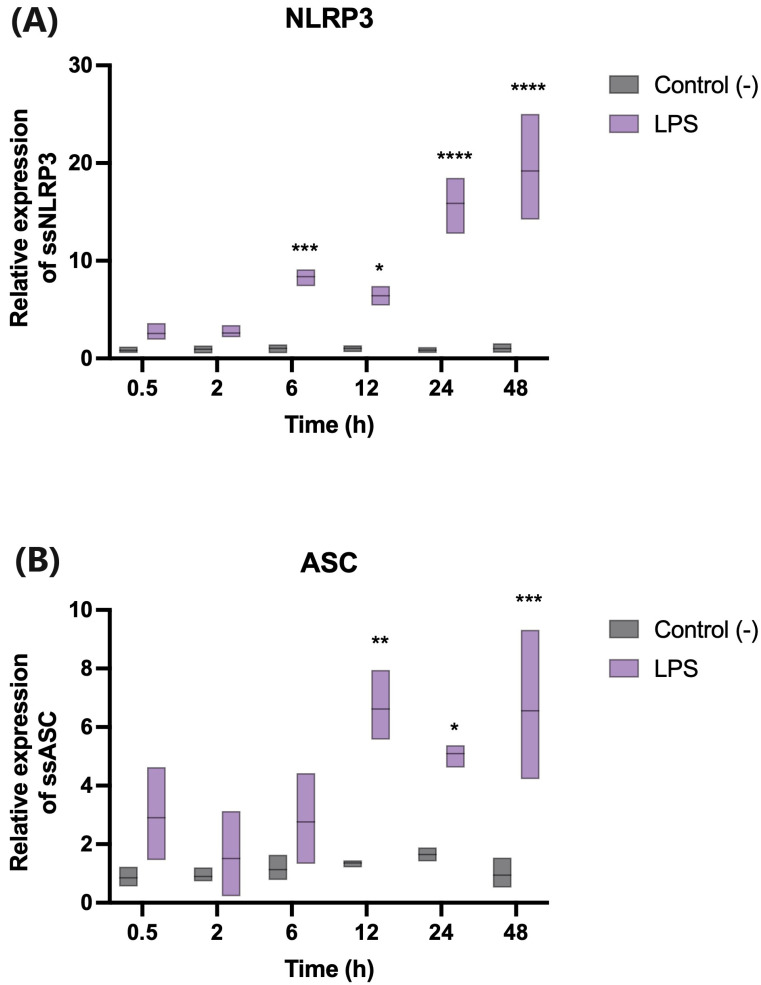
Transcript-expression analysis using qPCR in the SHK-1 cell line stimulated with LPS at different times to evaluate the presence of (**A**) NLRP3 and (**B**) ASC. Asterisks (*) indicate significant differences between treatments and control group. Bars represent mean values (S.E.). Symbols represent statistical difference by two-way ANOVA; *n* = 3. The differential differential analysis was compared between groups, with an adjusted *p* value of (* *p* < 0.05; ** *p* < 0.01; *** *p* < 0.001, **** *p* < 0.0001).

The NLRP3 transcription was up-regulated at all the times analyzed during the trial, which were 6 and 12 h after stimulation with LPS. Its over-expression was greater than six times. However, the maximum up-regulation was during the 24 h and 48 h after the stimulation with the LPS, reaching values over 15 times those of the control (Figure 4A).

The ASC transcription was also up-regulated at 0.5, 2, and 6 h in the three experimental conditions. However, there was a statistically significant increase in the cells stimulated with LPS at 12, 24, and 48 h (Figure 4B).

### 2.5. Transcriptome-Data Functional Annotation and Classification

**Figure 5:**
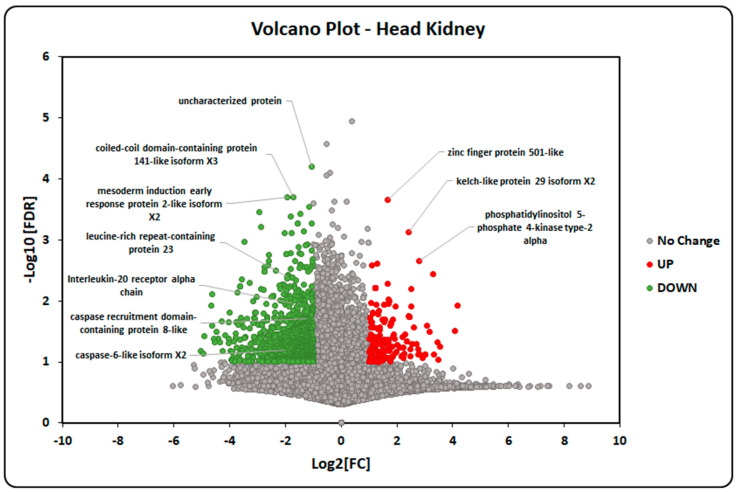
Volcano plot of the *Atlantic salmon* genes analyzed in the transcriptome data. Genes with skewed expression patterns between Parr (red) and smolt (green) salmon are shown. Green dots represent genes with significantly lower abundances, while red dots show genes with higher expression levels. Values on the x- and y-axes are log 2 fold change (FC) differences in the gene expression and negative log 10 of the corrected *p* values, respectively.

**Figure 6:**
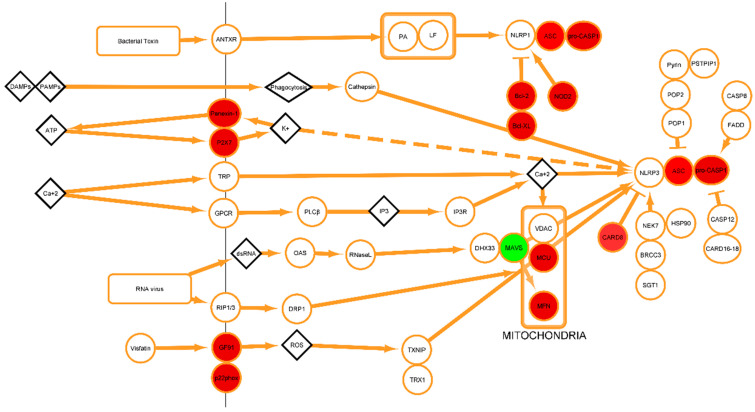
Representation of the *Atlantic salmon* inflammasome pathway genes analyzed in the transcriptome data. Down-regulated genes are shown in red, and up-regulated genes are shown in green.

Figure 5 and Figure 6 show that most the inflammasome pathway genes were down-regulated in the head kidney tissue from the smolt, including ASC, pro-CASP-1, leucine-rich repeat-containing protein, caspase-recruitment-domain-containing protein, interleukin-20 receptor, etc. Furthermore, Table 2 shows five other genes analyzed in the transcriptome: the C-X-C motif chemokine 10, apoptosis regulator bcl-2, apoptosis-associated speck-like protein containing a CARD, NLR-family member X1, and caspase-recruitment-domain-containing protein 8-like. All the genes in Table 2 showed a tendency to be consistently down-regulated in the smolt compared to the parr. However, not all the reads showed the same behavior. These genes were chosen due to their abrupt and consistent down-regulation in almost all the reads. Following this example, we created a cytoscape representation of the up- or down-regulated inflammasome genes (Figure S6). Figure 6 shows the NLRP3-associated genes (zoom-in of Figure S6). There are genes related to the early stages of innate immunity, such as *Panexin-1*, and *P2X7*, and others associated with ROS, such as GF91 and p22phox, as well as those associated with apoptosis, including Bcl-2, Bcl-XL, ASC, NOD2, pro-CASP1, MCU, MFN, and CARD8. All these were all down-regulated after the parr–smolt transformation. Finally, only MAVS (mitochondrial antiviral-signaling protein), which is essential for antiviral innate immunity, was up-regulated.

## 3. Discussion

The NLRP3-inflammasome assembly occurs in response to the detection of exogenous pathogens or endogenous cell damage [13], mainly through the activation of some NLRs. The NLRP3 acts as a sensor, ASC acts as an adapter, and pro-caspase-1 acts as an effector. Inflammasome formation results from the autoproteolytic activation of caspase-1, which also cleaves pro-inflammatory cytokines, such as pro-IL-1β/IL-18, into their mature forms to induce membrane blebbing, cell pyroptosis, and cytokine release [18].

In the present study, we identified homologous sequences for SsNLRP3, which has a similar structural organization to the main functional domains and tertiary structure of human NLRP3, except for a C-terminal PRYSPRY and SNTX domains (Figure 1 and Figure S2). These are involved in innate immune signaling, cytokine-signaling suppression, development, cell growth and retroviral restriction [33]. The LRRs act as pathogen sensors. Differences in the number of amino acids in the LRRs between human and SsNLRP3 have been identified; however, these differences may not be significant [34]. Studies carried out on mammals suggest that the lack of LRR domain in NLRP3 can still activate the canonical pathway of the inflammasome [35]. Recent studies with cryo-electron microscopy on mammalian NLRP3 have provided further details, including the structure of the inactivated NLRP3 complex [36], the cage that prevents premature activation by concealing the pyrin domains [37], the molecular basis of oligomerization [38], and the disk-shaped structure of the active oligomers of the NLRP3-inflammasome complex [39].

Our efforts to model the inactive NLRP3 complex from *Atlantic salmon* represent an initial stride towards obtaining more structural details from this commercially significant fish species, which is of particular importance for countries such as Norway, Chile, and Scotland, among others [40]. Despite the evolutionary distance between fish and mammals, we highlight the structural similarities between these complexes in this study. A three-dimensional model of the SsNLRP3 inflammasome was constructed, guided by the structure of the human NLRP3 inflammasome [41]. To assess the potential for inflammasome assembly, a docking analysis of the modeled *Atlantic salmon* NLRP3, ASC, and caspase-1 was performed. We propose an SsNLRP3-inflammasome model (Figure 4) based on a three-dimensional structure of human NLRP3 protein generated by Liu et al., 2020 [42] and a previously described human NLRP3 inflammasome [41,43,44]. According to the resulting model, the SsNLRP3 inflammasome might possess a structural arrangement similar to that of its human counterpart. The components of both complexes are bound by the same connectors, such as the PYD domain, with NLRP3 and ASC, and CARD, with caspase-1. However, it is important to note that the SsASC CARD2 only has a 29.5% identity and a 45.8% similarity against the hASC CARD sequence.

Additionally, we analyzed the functionality of the *NLRP3* and *ASC* genes involved in NLRP3-inflammasome activation. After the stimulation with the LPS, the SHK-1 cells exhibited increases in the expression of NLRP3 and ASC, which are associated with the activation of the NLRP3 inflammasome [13,23,45,46]. Elevated expressions of mRNA for inflammasome components (NLRP3, caspase-1, and ASC) have been described in various teleost fish species [19]. This phenomenon has also been observed in numerous tissues of the immune system, including those in the spleen, head kidney, gills, and intestines, in line with their regulatory functions in these organs [17,47].

Furthermore, it has been reported that the mRNA expression of inflammasome components (NLRP3, ASC, and caspase-1) varies in response to bacterial infections. For instance, in zebrafish (*Danio rerio*), a significant increase in *DrNLRP3*-gene expression was observed four hours post-infection with *Edwardsiella tarda* [47]. In Nile tilapia, the expression of the *NLRP3* gene initially decreased post-infection with *Streptococcus agalactiae*, and then increased and reached its peak level on the eighth day [48]. Similar patterns have been observed in other fish species following infection [19]. These increases in the transcriptional expression of the genes involved in the inflammasome align with our studies conducted on the SHK-1 cell line, stimulated at different time intervals with pathogen-associated molecular patterns (PAMPs), such as LPS. Under these conditions, significant increases in NLRP3 and ASC mRNA expression were observed, primarily at 24 and 48 h post-stimulation. These findings are consistent with expression analyses of TnNLRP3, TnASC, and Tncaspase-1 across various tissues (spleen, head kidneys, gills, and intestines) in *T. nigroviridis* fish post-infection with *V. parahaemolyticus* [19]. Significant increases in the expression levels of TnNLRP3 and TnASC were observed in the gills at 24 h. Moreover, peak expression levels were observed at 48 h in both the head kidneys and the intestines, and at 24 h in the spleen. Consequently, these results suggest that the *Atlantic salmon* NLRP3 inflammasome is closely related to the immune systems of other fish species and plays a significant role in the antibacterial immune response.

According to transcriptome analysis, we evaluated the SsNLRP3 expression and inflammasome-related genes during smoltification. Interestingly, many genes associated with the NLRP3 inflammasome were down-regulated in the smolt compared to the parr salmon. These expression changes can be anticipated by considering the high energy costs associated with osmoregulation and adaptation to the marine environment processes that occur during smoltification.

Even though parr–smolt transformation is one of the most relevant research areas in salmon biology, the immune-system changes in fish during this process often receives insufficient attention. In recent years, some research groups have started to present data that associate the increase in infectious diseases observed after the transfer to seawater with alterations in the immune response during smoltification [49,50]. Post-smolts show a weak viral response [51,52,53], and their skin barriers [54] and intestines [55] are weak during the first post-smolt period. Decreased plasma levels of lysozyme, IgM, and leukocytes have also been noted [56,57]. Furthermore, a study by Pontigo et al. [58], determined that NLRC5 decreases its expression during the parr–smolt transformation in *Atlantic salmon*’s three main immune organs, the head kidneys, spleen, and hindgut. Furthermore, it has been suggested that some SsNLR isoforms (NLRC3) may express differently towards a bacterial infection (*Piscirickettsia salmonis*) prior to smoltification [59].

In our transcriptome analysis, the NLRP3-inflammasome-pathway genes were found to be down-regulated in the head kidneys at the conclusion of the parr–smolt transformation. This was in line with our expectations, given the known decrease in immune-system function at the end of this transformation process in *Atlantic salmon* [49] (Supplementary Figure S6). This observed decrease in the expression of NLRP3-associated genes in smolt fish, which aligns with the physiological down-regulation of the immune system at the end of the parr–smolt transformation, not only provides the first in vivo description of these genes, but also allows us to speculate about their functional role in vivo. However, additional in vivo functional studies are required to definitively establish the role of the NLRP3 inflammasome in the immune response of *Atlantic salmon*.

## 4. Materials and Methods

### 4.1. Sequences and In Silico Structural Analysis

#### 4.1.1. Analysis of Sequences and Construction of the Phylogenetic Tree

*Salmo salar NLRP3*-gene sequence was retrieved from the Genbank database (accession number XP_013994834.1). A consensus phylogenetic tree was constructed for *NLRP3* gene products to infer phylogenetic relationships between related species. To this end, protein sequences from several species were extracted from the NCBI database and aligned using ClustalW tool included in the Geneious prime software (version 2021.1.1). The alignment was improved manually by removing the gaps. The phylogenetic tree was constructed using the MrBayes software version 3.2.7 for 100,000 generations and burned by 25% [60].

#### 4.1.2. Template Selection

NLRP3, ASC, and caspase-1 amino acid sequences of *Atlantic salmon* were retrieved from NCBI (accession numbers XP_013994834.1, ACI66706.1, and XP_014070034.1, respectively). Templates were selected based on the e-value of the BLAST search and its sequence identity with SsNLRP3, SsASC, and ssCaspase-1. Based on these criteria, the cryo-EM structure of human NLRP3 (PDB entry 6NPY) and the structure of full-length human ASC (PDB entry 2KN6) were selected as templates to model NLRP3 and ASC, respectively. In addition, the structure of human inhibitor interleukin-1 beta generation and human caspase-1 CARD (PDB entry 5FNA) were used as templates to model the caspase-1 CARD2 domain, whereas the remaining structures (p20 and p10 domain) were modeled using a crystal structure of human procaspase-1 zymogen domain (PDB entry 3E4C) as template.

#### 4.1.3. Homology Modeling of SsNLRP3, SsASC and SsCaspase-1

Homology models were built for SsNLRP3, SsASC, and SsCaspase-1 using the crystal structure of the protein templates mentioned above. Models were built using MODELLER v9.25 and SWISS-MODEL server [61,62,63]. Subsequently, the stereo-chemical quality of the models was assessed using PROCHECK (overall average G factor and Ramachandran plot) and QMEAN [64,65]. Loop regions of NLRP3 y caspase-1 were refined using the Mod Loop server [66,67]. The resulting models were subjected to energy-minimization cycles until convergence (Supplementary Figure S1) and equilibration for 20 ns to relax the conformation of side chains and prevent conformational tension generated by the homology model. All calculations were performed using NAMD (Nanoscaled Molecular Dynamics, version 2.9) and CHARMM force field [68,69]. Models were visualized and rendered using Visual Molecular Dynamics (VMD, version 1.9) [70].

#### 4.1.4. Molecular Docking between SsNLRP3, SsASC and SsCaspase 1

*Atlantic salmon* inflammasome complex was predicted using HADDOCK 2.2 protein-protein docking web server [71] using human NLRP3 inflammasome as guide (dos Santos et al., 2012) to analyze the binding interface between their components. To this end, we first identified the binding interface between SsNLRP3 and SsASC and then that between SsNLRP3/SsASC and ssCaspase-1 based on the HADDOCK score. In HADDOCK [72], the scoring function consists of a linear combination of various energies and buried surface area, which differs for the three docking stages: rigid and semi-flexible body refinement, and explicit solvent refinement (water).

$$Score=w_{1}E_{vdw}+w_{2}E_{elec}+w_{3}E_{air}+w_{4}E_{rg}+w_{5}E_{sani}+w_{6}E_{vean}+w_{7}E_{pcs}+w_{8}E_{dani}+w_{9}E_{cdih}+w_{10}E_{sym}+w_{11}BSA+w_{12}dE_{int}+w_{13}E_{desol}$$

where *E_vdw_* is the van der Waals intermolecular energy, *E_elec_* is the electrostatic energy, *E_air_* is the distance-restraints energy, *E_rg_* is the radius of gyration-restraint energy, *E_sani_* is the direct RDC-restraint energy, *E_vean_* is the intervector-projection-angle-restraints energy, *E_pcs_* is the pseudo-contact-shift-restraints energy, *E_dani_* is the diffusion-anisotropy energy, *E_cdih_* is the dihedral-angle-restraints energy, *E_sym_* is the symmetry-restraints energy, *BSA* is the buried surface area, *dE_int_* is the binding energy (E_total_complex_ − Sum[E_total_components_]), and *E_desol_* is the desolvation energy.

### 4.2. Transcript-Expression Analysis

#### 4.2.1. Experiment Preparation

The salmon head kidney (SHK-1) cell line was purchased from the European Collection of Authenticated Cell Cultures (ECACC). It was cultured at 18 °C in 75 cm^2^ tissue-culture-treated flasks (Costar) in L-15 medium (500 mL with 300 mg/L L-glutamine) supplemented with 500 mL of gentamicin sulfate (50 mg/mL in dis- tilled water), 365 mL of 2-mercaptoethanol (55 mM in Dulbeco’s phosphate-buffered saline), and 5% fetal bovine serum (FBS) [73].

For use of the salmon head kidney (SHK-1) cell line was used, cells were counted (5 × 10^7^ cells) and seeded at a final volume of 2 mL per plate in 6-well cell-culture plates [74]. Cell cultures were then supplemented with fetal bovine serum (FBS, 0.1%) and stimulated in duplicate wells with LPS (10 µg/mL) or PBS as a control. Sampling was performed at 0.5, 2, 6, 12, 24, and 48 h. The supernatant was removed, and 500 µL of Trizol was added to each well, and then transferred to a 1.7 mL Eppendorf tube and immediately frozen at −80 °C [75].

#### 4.2.2. qPCR Analyses

Total RNA was extracted with the TRIzol reagent (Invitrogen, Carlsbad, CA, USA) following the manufacturer’s instructions. Samples were treated with amplification-grade DNase I (1 U μg^1^ RNA, Invitrogen). The SuperScript III RNase H Reverse Transcriptase platform (Invitrogen) was synthesized from first-strand cDNA from 1 μg of total RNA using the oligo-dT18 primer at 50 °C for 60 min. Reactions were carried out using an AriaMx Real-Time PCR System. Analyses of qPCR used cDNA diluted to 100 ng as a template and Brilliant SYBR^®^ Green qPCR Master Mix (Stratagene, Carlsbad, CA, USA). 

All reactions were performed in triplicate. The PCR protocol applied was as follows: 95 °C for 10 min, followed by 40 cycles at 95 °C for 15 s, 60 °C for 1 m, and finally 95 °C for 15 s. Melting-curve analysis of amplification products was performed at the end of each PCR to confirm the detection and sequence amplification of only one product. All genes were analyzed using the comparative Ct (∆∆Ct) method [76]. Data are expressed as the relative mRNA expression normalized to the housekeeping gene (*18S*), according to the values obtained for unstimulated cells (Table 3). The quantity, purity, and quality of the isolated RNA were measured on the TapeStation 2200 (Agilent Technologies Inc., Santa Clara, CA, USA) using RNA ScreenTape according to the manufacturer’s instructions. All RNAs had an RIN > 7.0. All data are given in terms of relative expression and are expressed as means ± standard errors of the means (SE). The PCR efficiencies were calculated according to Equation (1), providing efficiencies between 95% and 105%:

(1) $$E=10^{-(1/slope)}-1\;$$

### 4.3. Gene-Expression and Transcriptome Analyses

#### 4.3.1. Ethics Statement

The study adhered to animal-welfare procedures suggested by the National Research and Development Agency (Agencia Nacional de Investigación y Desarrollo ANID) in the Chilean government. Furthermore, all experimental protocols involving fish were approved by the bioethical committees of the Austral University of Chile (N° 318/218).

#### 4.3.2. Fish and Sampling: Animals

*Atlantic salmon* (*Salmo salar*), parr, and smolt were provided by Calabozo Salmon Farm, Chile (Mowi, Chile). In each sampling, groups of five salmon were euthanized with 1 mL/L 2-phenoxyethanol (Fluka-77699-500 ML), followed by spinal sectioning. Head-kidney tissue was quickly dissected out and frozen immediately on liquid nitrogen. All samples were stored at −80 °C until use.

#### 4.3.3. Transcriptome Sequencing and De Novo Assembly

Total RNA was extracted from frozen head-kidney tissues using Nucleo spin tissue (Macherey-Nagel^®^, Valencienner, Duren,· Germany). Total RNA was treated with DNAse I (Thermo Scientific, Carlsbad, CA, USA). The quantity, purity, and quality of isolated RNA were measured in the TapeStation 2200 (Agilent Technologies Inc., Santa Clara, CA, USA) using RNA ScreenTape, according to the manufacturer’s instructions. Head-kidney RNA samples from five fish with RIN > 8.0 were pooled and used for library preparation. Subsequently, double-stranded cDNA libraries were constructed by using TruSeq RNA Sample Preparation Kit v2 (Illumina^®^, San Diego, CA, USA) with RNA pools of parr and smolt. Two biological replicates for each sample pool were sequenced by MiSeq (Illumina^®^, San Diego, CA, USA) platform using sequenced runs at the AUSTRAL-*omics*, Universidad Austral de Chile.

#### 4.3.4. Transcriptome Analysis of *Atlantic salmon* Smoltification

A comparative analysis of different genes’ expression related to the inflammasome pathways in the head kidneys of *Atlantic salmon* was carried out using transcriptomics data in both parr and smolt stages. Each stage has two different cDNA libraries. The genes were selected according to the NOD-like receptor signaling pathway, *Salmo salar* (*Atlantic salmon*), taken from KEGG pathway (https://www.genome.jp/kegg-bin/show_pathway?sasa04621) (accessed on 19 September 2020).

Alignment and annotation of Fastq sequences were carried out using Hisat2 and Htseq counts, respectively [77,78,79]. To this end, the *Atlantic salmon* genome assembly was taken from GenBank (Accession number GCA_000233375.4). Later, data were imported into Excel. Changes in expression levels between parr and smolt were established as a parr/smolt ratio. Finally, a visual representation of the inflammasome genes, identified through transcriptome analysis, was created using Cytoscape [80]. Additionally, RNA-Seq results were visualized and analyzed with Volcano Plot using R package.

## 5. Conclusions

In conclusion, we constructed a homology model for the *Atlantic salmon*’s NLRP3-inflammasome complex using the 3D structures of 6NPY, 2KN6, 5FNA, 1DNG, and 3E4C. The model was subsequently validated using a Ramachandran plot, revealing structural rearrangements closely akin to those of the human NLRP3 inflammasome.

We tested the functionality of the NLRP3 inflammasome both in vitro and in vivo. Initially, we stimulated SHK-1 cells with LPS, leading to an increase in the expression of genes involved in inflammasome activation. This suggests that the NLRP3 inflammasome is involved in pathogen defense by activating the immune response. Finally, our in vivo findings showed that many genes associated with the NLRP3 inflammasome are down-regulated after parr–smolt transformation, during which salmonids adapt to living in seawater. This down-regulation correlates with the temporary weakening of the immune system observed. These findings provide valuable insights into the *Atlantic salmon*’s immune response and the potential role of the NLRP3 inflammasome in disease resistance.

## Figures and Tables

**Table 1:** Binding sites of inflammasome components.

Protein	Domain	Binding Site
SsNLRP3	PYD	Ser24, Ser25, Gln26, Leu28, Lys29, Trp33, Lys36, Ser49, Glu52, Ala54, Lys55, Glu57, Thr59, Arg56
SsASC	PYD	Leu10, Ala11, Leu13, Glu14, Lys15, Leu16, Asp17, Lys18, Glu45, Asp46, Ala47, Ser48, Arg49, His50, Asp80
	CARD2	Pro133, Ile134, Asp136, Gly137, Leu138, Tyr139, Gln140, Lys141, Met143, Lys142, Asp146, Ile183, Gln188, Ser189, Leu191
SsCaspase-1	CARD2	Asp3, Ser7, Arg10, Lys11, Ile14, Asp15, Glu39, Ser42, Glu52, Arg55, Cys56, Asp59, Met60, Arg62, Lys63, Gly65, Ser66

Amino acids colored in blue and red are involved in H-bonds and hydrophobic contacts, respectively.

**Table 2:** Genes analyzed in the transcriptome during smoltification in *Atlantic salmon*.

Head Kidney A RPM Parr/Smolt	Head Kidney B RPM Parr/Smolt	RNA Name
0.37	0.41	C-X-C motif chemokine 10 (C-X-C motif chemokine 10 precursor)
↓	↓	
0.00	0.18	Apoptosis regulator Bcl-2
0.23	1.41	Apoptosis regulator Bcl-2
0.00	1.07	Apoptosis regulator Bcl-2
0.72	0.34	Apoptosis regulator Bcl-2
↓	↓	
1.00	1.00	Apoptosis-associated speck-like protein containing a CARD
0.79	0.46	Apoptosis-associated speck-like protein containing a CARD
0.74	1.00	Apoptosis-associated speck-like protein containing a CARD
1.09	0.79	Apoptosis-associated speck-like protein containing A CARD
↓	↓	
0.61	0.74	NLR family member X1 isoform X2
↓	↓	
0.42	0.99	Caspase-recruitment-domain-containing protein 8-like
0.23	1.38	Caspase-recruitment-domain-containing protein 8-like isoform X2
0.00	0.36	Caspase-recruitment-domain-containing protein 8-like isoform X2
0.34	0.43	Caspase-recruitment-domain-containing protein 8-like
0.14	1.19	Caspase-recruitment-domain-containing protein 8-like isoform X1
0.00	2.75	Caspase-recruitment-domain-containing protein 8-like
0.00	0.61	Caspase-recruitment-domain-containing protein 8-like
0.00	1.00	Caspase-recruitment-domain-containing protein 8-like
↓	↓	

A and B are two different pools of RNA (extracted from five fish).

**Table 3:** Genes analyzed in qPCR.

Gene		Sequence (5′ → 3′)	Accession Number	Efficiencies (%)	Amplicon Size
*SsNLRP3*	Forward	AGAGGGTCTATCTGGGCCTG	XP_013994834.1	113.8	110
	Reverse	CTTTACGCCCTCCTGTCCTG			
*SsASC*	Forward	GGTAACATCGGGTGCTGCTA	ACI66706.1	105.9	127
	Reverse	CCTGGCTCACTCTGTCGATC			
*18S*	Forward	GTCCGGGAAACCAAAGTC	XR_006760234.1	102.3	119
	Reverse	TTGAGTCAAATTAAGCCGCA			

## Data Availability

Not applicable.

## Supplementary Materials

The following supporting information can be downloaded at: https://www.mdpi.com/article/10.3390/ijms241914556/s1.

## Abbreviations

ASC, apoptosis-associated speck-like protein containing a CARD; BIR, Baculovirus IAP Repeat domain; BSA, buried surface area; BWA, Burrows–Wheeler Alignment; CARD, N-terminal recruiter domain; CHARMM, Chemistry at HARvard Macromolecular Mechanics; DAMP, damage-associated molecular patterns; LPS, lipopolysaccharide; LRR, leucine-rich repeat; NLRP, nucleotide-binding oligomerization domain and leucine-rich repeat protein; MAVS, mitochondrial antiviral-signaling protein; NOD, nucleotide-binding oligomerization domain; PAMP, pathogen-associated molecular pattern; PRRs, pattern-recognition receptors; PDB, protein-data bank; RDC, residual dipolar coupling; PYD, N-terminal pyrin domain; qPCR, quantitative polymerase chain reaction; ROS, reactive oxygen species; RPM, reads per million mapped reads; SHK-1, salmon head kidney; VMD, visual molecular dynamics.
