# Supplementary material for: Revealing the Salmo salar NLRP3 Inflammasome: Insights from Structural Modeling and Transcriptome Analysis
Source: Int J Mol Sci. 2023 Sep 26;24(19):14556. doi: 10.3390/ijms241914556 (PMC10572965; doi:10.3390/ijms241914556)
Supplement: Supplementary file 1 [file ijms-24-14556-s001.zip › ijms-2603603-supplementary/Material Suplementario/Supplementary Material NLRP3.pdf]

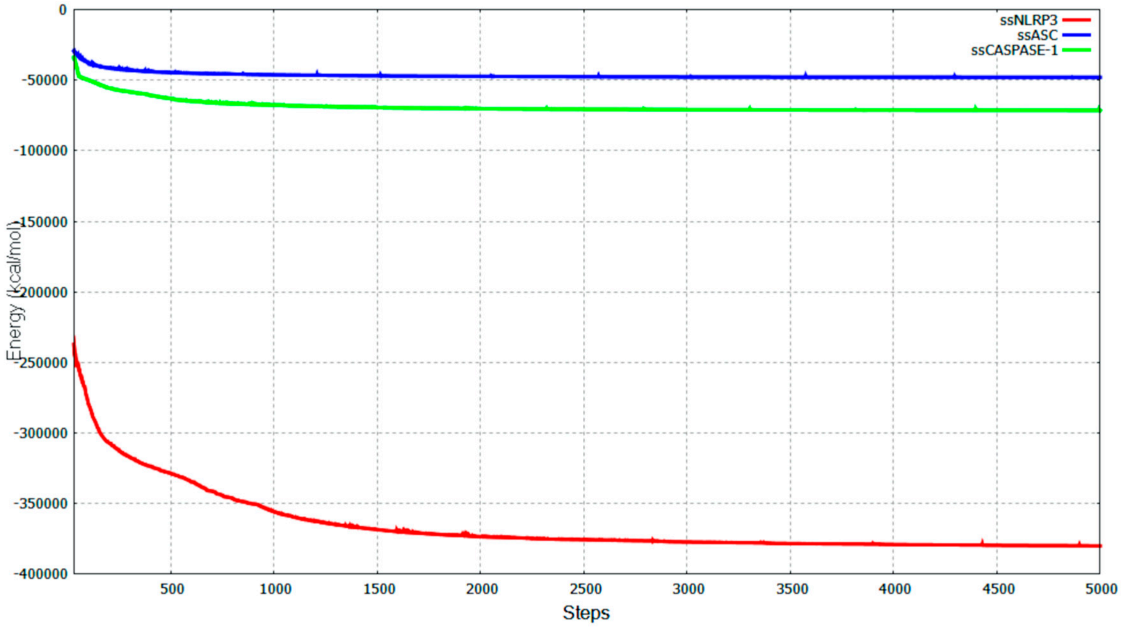

Supplementary Figure S1. Energy minimization of (a) ssNLRP3, (b) ssASC, and (c) ssCaspase-1 for 5000 steps.

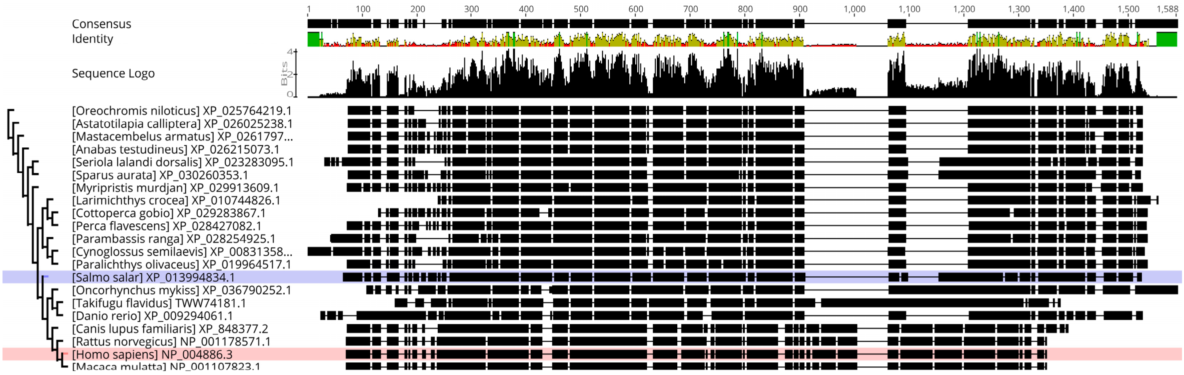

Supplementary Figure S2. Sequence multiple alignment of NLRP3 from different organisms.

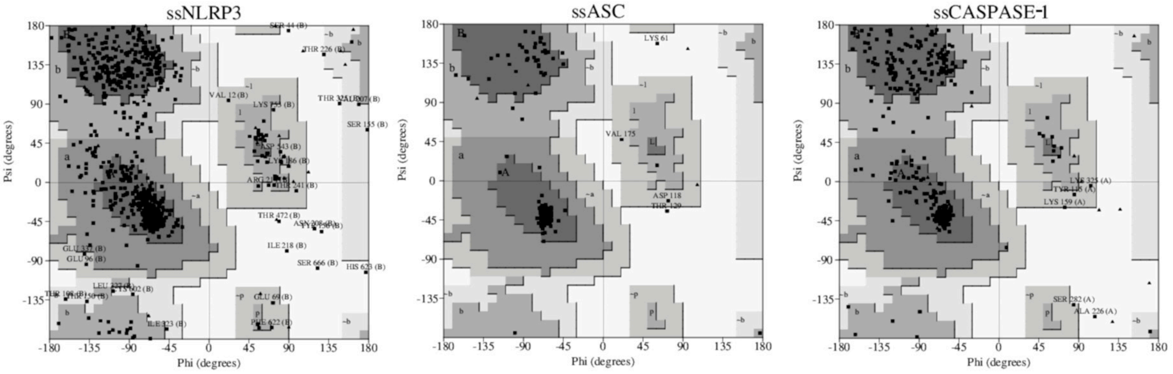

Supplementary Figure S3. Conformational analyses of inflammasome components. Ramachandran plots for (a) SsNLRP3, (b) SsASC, and (c) SsCaspase-1, produced with PROCHECK. The core regions are shown in dark gray, allowed regions in gray, and generously allowed regions in light gray. The disallowed regions are shown in white.

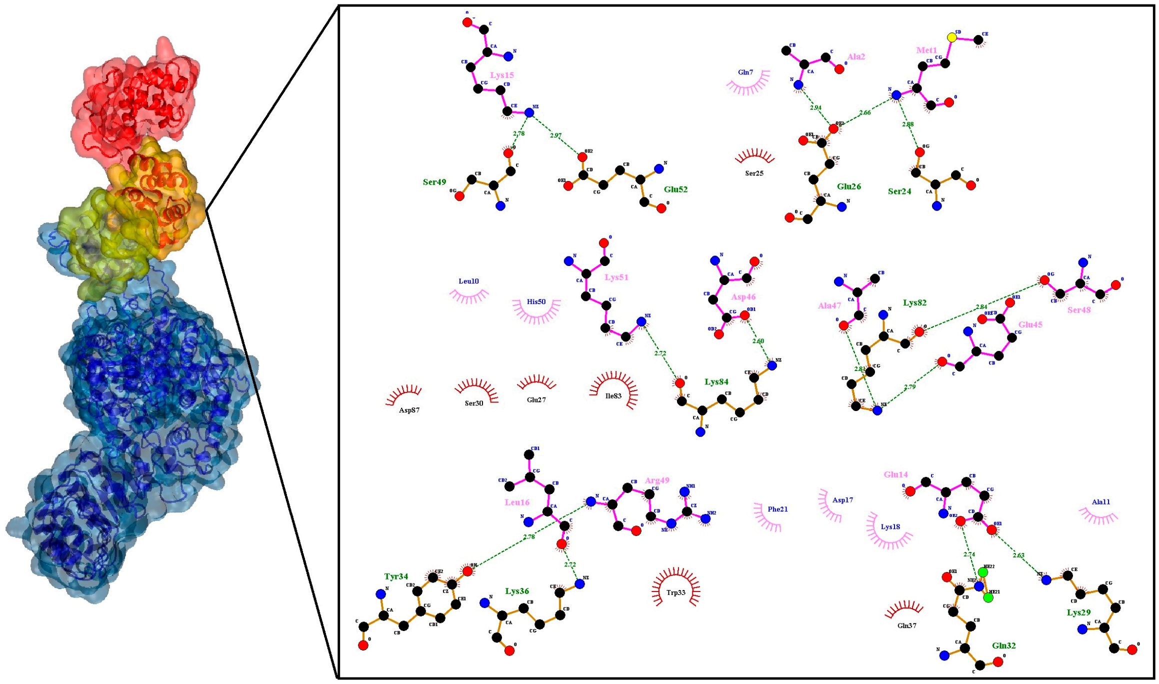

Supplementary Figure S4. Visualization of binding sites of the SsNLRP3/SsASC complex.

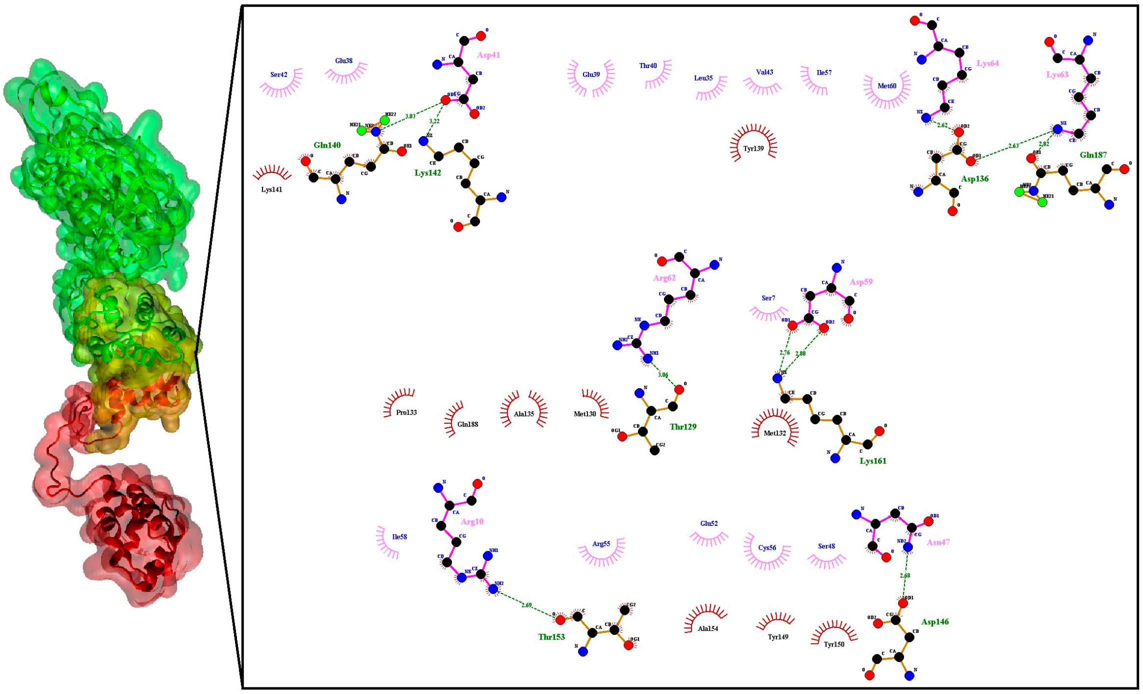

Supplementary Figure S5. Visualization of binding sites of the SsASC/SsCaspase-1 complex.

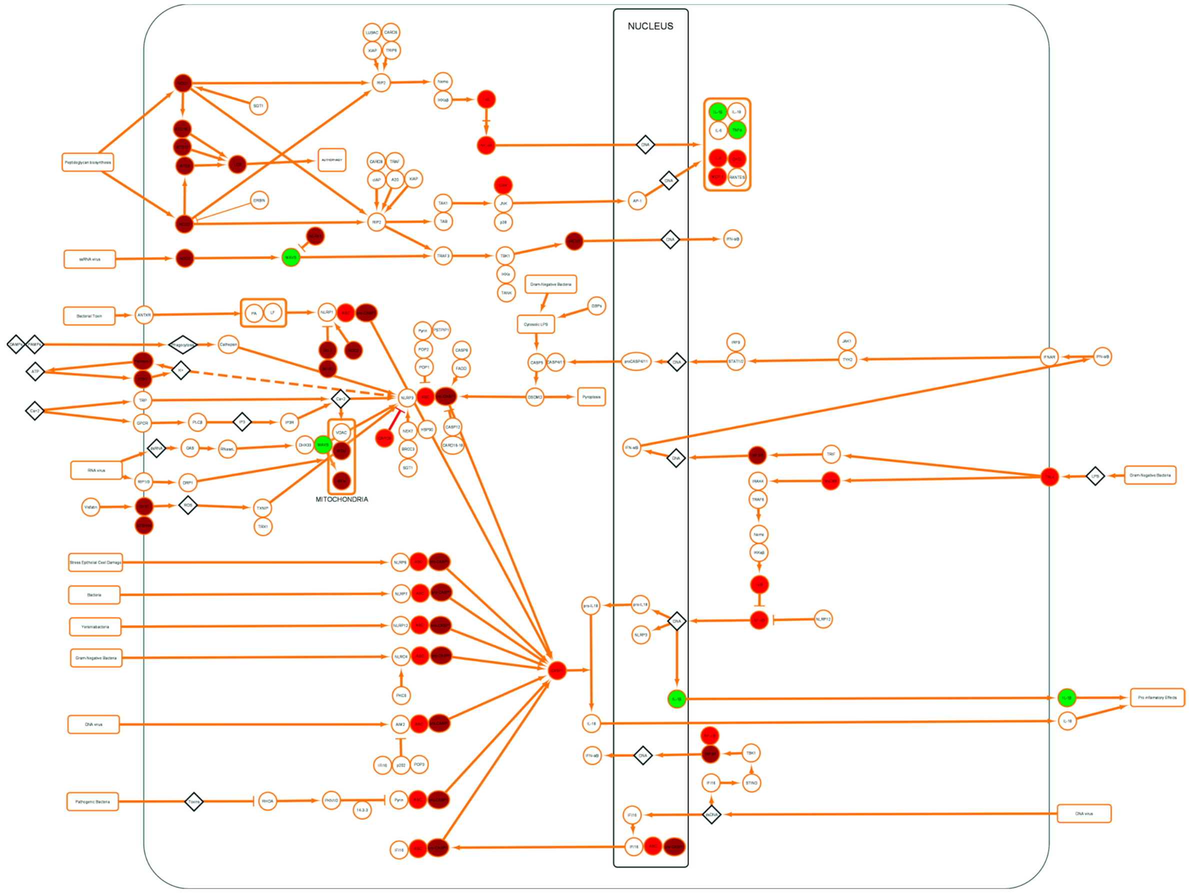

Supplementary Figure S6. Representation of all the genes analyzed in the transcriptome data. Downregulated genes are shown in red, and upregulated genes are shown in green.
